# Effectiveness of Cognitive Behavioral Therapy for Depression in Patients Receiving Disability Benefits: A Systematic Review and Individual Patient Data Meta-Analysis

**DOI:** 10.1371/journal.pone.0050202

**Published:** 2012-11-29

**Authors:** Shanil Ebrahim, Luis Montoya, Wanda Truong, Sandy Hsu, Mostafa Kamal el Din, Alonso Carrasco-Labra, Jason W. Busse, Stephen D. Walter, Diane Heels-Ansdell, Rachel Couban, Irene Patelis-Siotis, Marg Bellman, L. Esther de Graaf, David J. A. Dozois, Peter J. Bieling, Gordon H. Guyatt

**Affiliations:** 1 Department of Clinical Epidemiology and Biostatistics, McMaster University, Hamilton, Canada; 2 Department of Dentistry, Santo Tomas University, Bogota D.C., Colombia; 3 McMaster Integrative Neuroscience Discovery & Study program, McMaster University, Hamilton, Canada; 4 Department of Oncology, McMaster University, Hamilton, Canada; 5 Ain Shams University, Faculty of Medicine, Cairo, Egypt; 6 Evidence-Based Dentistry Unit, Faculty of Dentistry, University of Chile, Santiago, Chile; 7 Department of Anesthesia, McMaster University, Hamilton, Canada; 8 Department of Mathematics and Statistics, McMaster University, Hamilton, Canada; 9 Psychiatry & Behavioural Neurosciences, McMaster University, Hamilton, Canada; 10 Mood Disorders program, St. Joseph's Healthcare, Hamilton, Canada; 11 National Disability Services, Policy & Procedure department, Sun Life Financial, Toronto, Canada; 12 Department of Medical Psychology and Psychotherapy, Erasmus MC University Medical Center, Rotterdam, The Netherlands; 13 Department of Psychology, University of Western Ontario, London, Canada; 14 Department of Medicine, McMaster University, Hamilton, Canada; University of York, United Kingdom

## Abstract

**Objectives:**

To systematically summarize the randomized trial evidence regarding the relative effectiveness of cognitive behavioural therapy (CBT) in patients with depression in receipt of disability benefits in comparison to those not receiving disability benefits.

**Data Sources:**

All relevant RCTs from a database of randomized controlled and comparative studies examining the effects of psychotherapy for adult depression (http://www.evidencebasedpsychotherapies.org), electronic databases (MEDLINE, EMBASE, PSYCINFO, AMED, CINAHL and CENTRAL) to June 2011, and bibliographies of all relevant articles.

**Study Eligibility Criteria, Participants and Intervention:**

Adult patients with major depression, randomly assigned to CBT versus minimal/no treatment or care-as-usual.

**Study Appraisal and Synthesis Methods:**

Three teams of reviewers, independently and in duplicate, completed title and abstract screening, full text review and data extraction. We performed an individual patient data meta-analysis to summarize data.

**Results:**

Of 92 eligible trials, 70 provided author contact information; of these 56 (80%) were successfully contacted to establish if they captured receipt of benefits as a baseline characteristic; 8 recorded benefit status, and 3 enrolled some patients in receipt of benefits, of which 2 provided individual patient data. Including both patients receiving and not receiving disability benefits, 2 trials (227 patients) suggested a possible reduction in depression with CBT, as measured by the Beck Depression Inventory, mean difference [MD] (95% confidence interval [CI]) = −2.61 (−5.28, 0.07), p = 0.06; minimally important difference of 5. The effect appeared larger, though not significantly, in those in receipt of benefits (34 patients) versus not receiving benefits (193 patients); MD (95% CI) = −4.46 (−12.21, 3.30), p = 0.26.

**Conclusions:**

Our data does not support the hypothesis that CBT has smaller effects in depressed patients receiving disability benefits versus other patients. Given that the confidence interval is wide, a decreased effect is still possible, though if the difference exists, it is likely to be small.

## Introduction

Major Depressive Disorder (henceforth referred to as depression) results in immense human suffering and an enormous socioeconomic burden. Depression accounts for 11% of disability worldwide and an estimated productivity loss of $17 to $44 billion in the USA [1], [2]. Depression is expected to become the second leading cause of disease burden worldwide by the year 2020 [3].

The National Institute for Health and Clinical Excellence (NICE) in the UK recommends that health care professionals provide pharmacological treatments and/or high-intensity psychological interventions for individuals suffering from depression. Pharmacological treatments may accelerate recovery from depression, particularly when symptoms are severe [4] and, over the last few decades, their use has increased dramatically in Western nations [5], [6]. NICE guidelines suggest psychological therapies should be offered to individuals suffering from persistent subthreshold symptoms of depression, mild to moderate depression, and those with a high risk of relapse or those declining pharmacological treatment for severe depression [5], [6].

Cognitive Behavioral Therapy (CBT) is a common non-pharmacological treatment for depression [5], [7]. CBT is based on three fundamental propositions: cognitive activity affects behavior, cognitive activity can be monitored and altered, and desired behavior change may be affected through cognitive change [7]. Twelve systematic reviews evaluating CBT in individuals suffering from depression have demonstrated that CBT reduces depressive symptoms [8], [9], [10], [11], [12], [13], [14], [15], [16], [17], [18], [19], with the most current and rigorous meta-analysis reporting a pooled standardized mean difference (SMD) of 0.69 (95% confidence interval [CI] of 0.59 to 0.79) [13].

In North America, depression is one of the most frequent reasons for receiving disability benefits [20], [21], and disability claims for mental health disorders incur greater costs compared to other disorders [22]. In those receiving disability benefits, individuals suffering from mental health disorders require more treatment and have greater difficulty returning to work than those suffering from other conditions [23]. Although CBT is one of the most frequently reimbursed therapies by insurers, its utilization by insurance companies still remains relatively low at approximately 3% for short-term disability claimants and 15% for long-term disability claimants [24].

CBT may be less effective, or ineffective, in patients receiving disability benefits, because their circumstances or psychological status may interfere with its successful implementation [25]. This may also be associated with the compensation process [26], secondary gain from financial benefits (benefits of assuming a sick role) [27], or the adversarial nature of litigation [28]. A recent meta-analysis of 129 studies in surgical populations that found a substantially greater risk of an unsatisfactory outcome (functional, quality of life, pain and patient satisfaction) after surgery in compensated patients (odds ratio [95% CI] = 3.79 [3.28 to 4.37]) provides indirect evidence for this hypothesis [29]. The effectiveness of CBT for depression in patients receiving disability benefits has received little attention.

### Objectives

The purpose of our study was to perform a systematic review and an individual patient data meta-analysis of all randomized controlled trials (RCTs) that compared the effectiveness of CBT to minimal/no treatment, or care-as-usual, in patients with depression receiving versus those not receiving disability benefits.

### Questions

In adult patients with depression, is there a difference in the effect of CBT on depression between those receiving disability benefits compared those not receiving disability benefits?

## Methods

We used the PRISMA guidelines [30] to report our findings.

### Protocol and registration

We developed a protocol prior to conducting the study but did not register it.

### Eligibility criteria

Eligible studies met the following criteria: 1) random allocation of adult patients to CBT or a control arm consisting of minimal/no treatment, treatment as usual (TAU) or pharmacotherapy if it was equally balanced in the treatment groups (e.g. CBT plus pharmacotherapy versus pharmacotherapy alone), and 2) inclusion of patients with depression, classified as Major Depressive Disorder by any edition of the Diagnostic and Statistical Manual (DSM), International Classification of Diseases (ICD), Research Diagnostic Criteria (RDC) or other diagnostic system [31].

### Information sources

We identified all relevant RCTs from a database of randomized controlled trials and comparative studies examining the effects of psychotherapy for adult depression (http://www.evidencebasedpsychotherapies.org) [32]. This database consisted of 281 trials and was identified from searching the following electronic databases in all languages: PUBMED, EMBASE, PsycINFO and Cochrane Central Register of Controlled Trials, from inception until January 1, 2011 [11]. In addition to the 281 trials, we updated the search with the assistance of an experienced academic librarian (RC) until June 13, 2011 for each electronic database, and also searched AMED and CINAHL. We hand searched the reference lists of all relevant RCTs for additional eligible trials.

### Search

Our search strategy including keywords and MESH headings are provided in Appendix A.

### Study selection

Three teams of reviewers (SE, SH, LM, WT, MK, ACL) worked in pairs and screened titles and abstracts of identified citations, independently and in duplicate, using a standardized, pilot-tested screening form. The same reviewers independently applied eligibility criteria to the full text of potentially eligible studies. One psychiatrist (IPS) and one psychologist (RM), blinded to study results, independently reviewed and confirmed eligibility of therapies that were not explicitly described by trial authors as CBT. We measured agreement for the full text review stage, and interpreted the agreement statistics using the guidelines proposed by Landis and Koch [33]. Kappa values of 0 to 0.20 represented slight agreement, 0.21 to 0.40 fair agreement, 0.41 to 0.60 moderate agreement, 0.61 to 0.80 substantial agreement, and greater than 0.80 almost perfect agreement.

Reviewers grouped eligible articles into one of the four categories: (i) studies that did not explicitly state if they included or excluded patients receiving disability benefits, (ii) studies that explicitly excluded patients receiving disability benefits, (iii) studies that explicitly included patients receiving disability benefits but did not separately report outcomes based on receipt of disability benefits, and (iv) studies that explicitly included patients receiving disability benefits and reported outcomes separately based on receipt of disability benefits. Disability benefits were defined as wage replacement benefits administered by a third party (e.g. insurer).

### Contacting authors of eligible studies

We identified 88 studies in category i, 4 in category iii, and none in either category ii or iv. Contact information was not reported and not available through an Internet search for authors of 22 (24%) trials. We attempted to contact authors of the remaining 70 trials by email and requested information on whether they had an eligibility stipulation for disability status. If authors included patients on disability benefits, we requested their trial data to facilitate an individual patient data meta-analysis (IPDMA). To maintain patient confidentiality, authors removed any personal identifiers from their dataset prior to transferring it to our center. We clarified uncertainties or discrepancies in the data sets with the study authors and combined individual patient data for variables that were similar across the trials. Based on authors' replies, we classified studies into four groups: (A) those that did have some data specific to patients on disability benefits, (B) those that confirmed that they had no patients on disability benefits, (C) those that did not have an eligibility criterion for disability status and did not collect information on disability status, and (D) unknown or did not respond.

### Data collection process

Using piloted standardized forms and a detailed instruction manual to extract data, the same teams of reviewers extracted data, independently and in duplicate, from studies in groups A and B. We did not abstract data from groups C and D.

Data abstracted included patient characteristics, treatment effect on depression, frequency and timing of follow-up, details of depression (including diagnostic classification system used, severity of depression, and duration of depression), and CBT intervention details (including the type of CBT administered, expertise of providers administering CBT, and frequency of CBT). Reviewers abstracted data from the following study arms: CBT, TAU and minimal or no treatment. Data comparing CBT only to active comparators were not abstracted, unless the active comparator was equally balanced between both the treatment and control group.

### Risk of Bias in individual studies

Using a modified Cochrane risk of bias instrument, reviewers assessed risk of bias for each eligible trial on the following domains: sequence generation; allocation concealment; blinding of participants, investigators, data collectors, outcome assessors, and data analysts; incomplete outcome data; selective outcome reporting; and other sources of bias (e.g. bias of study design, trial stopped early, extreme baseline imbalance, and fraudulent trial) [34], [35]. Reviewers used response options of “definitely yes”, “probably yes”, “probably no”, and “definitely no” with definitely and probably yes ultimately assigned high risk of bias and probably and definitely no assigned low risk of bias [35]. The reviewers resolved disagreements by discussion, and an arbitrator (JWB) adjudicated any remaining conflicts.

### Synthesis of results

For our IPDMA, we compared the effects (mean difference) of CBT on depression, measured by the most commonly reported instrument [Beck Depression Inventory (BDI–II)], in patients receiving disability benefits versus patients not receiving disability benefits. We used a one-stage method [36], and included the following variables in our model: study arm, receipt of disability benefits, interaction term of study arm and receipt of disability benefits, trial as a categorical variable, age and baseline BDI–II score. To guard against multiplicity of data [37], we used the most common follow-up time point of 3 months for our analysis.

Our secondary analyses evaluated whether there were differences in patients not in receipt of disability benefits between trials that included patients in receipt of disability benefits (group A) and trials with aggregate data that did not include patients receiving disability benefits (group B). We compared the following: 1) the effects of CBT between group A and B; 2) the effects of CBT between group A and B that compared CBT plus pharmacotherapy versus pharmacotherapy alone; 3) the effects of CBT between group A and B that compared CBT to TAU.

For our secondary analyses, we used the 2-stage method [38]. In the first stage, we aggregated the IPD data of the patients not receiving disability benefits in group A and in the second stage, pooled the aggregate data of studies in group A and B using a random-effects model.

We used the means and standard deviations (SDs) of the end of study scores for our secondary pooled analyses. To pool data across trials and to facilitate interpretation for clinicians and other stakeholders, we calculated the mean difference (MD) and its associated 95% confidence interval (CI) of the natural units of the most familiar instrument across trials, the BDI–II. For this calculation, we used the following formulas to convert mean estimates (M) and standard deviations (SD) into the scale of the most familiar instrument: M_A_ = (M_B_ - L_B_) (R_A_/R_B_)+L_A_ and SD_A_ = SD_B_ (R_A_/R_B_)+L_A_, where A represented the most familiar instrument and B represented the alternative instrument, L_A_ and L_B_ represent the lower range of instrument A and B respectively, and R_A_ and R_B_ represented the ranges for instruments A and B respectively [39].

We examined heterogeneity using both a chi-squared test and the I^2^ statistic [40]. Heterogeneity defined by an I^2^ of 0% to 40% was interpreted as ‘might not be important’, 30% to 60% as ‘moderate heterogeneity’, 50% to 90% as ‘substantial heterogeneity’, and 75% to 100% as ‘considerable heterogeneity’ [40]. We generated the following a priori hypotheses to explain variability between studies in our secondary analyses: studies using in-person CBT will have greater effects than studies using computer administered-CBT, and studies with high risk of bias will demonstrate larger effects compared to studies with low risk of bias.

We performed analyses using SPSS version 20 and the Cochrane Collaboration Review Manager software (RevMan version 5.1.2).

## Results

### Study selection

We screened 977 citations and retrieved 421 articles in full text; 329 studies did not meet inclusion criteria and 92 trials were deemed eligible. The kappa (95% CI) chance-corrected agreement on assessing full text eligibility was 0.74 (0.66 to 0.81), representing substantial agreement.

**Figure 1:**
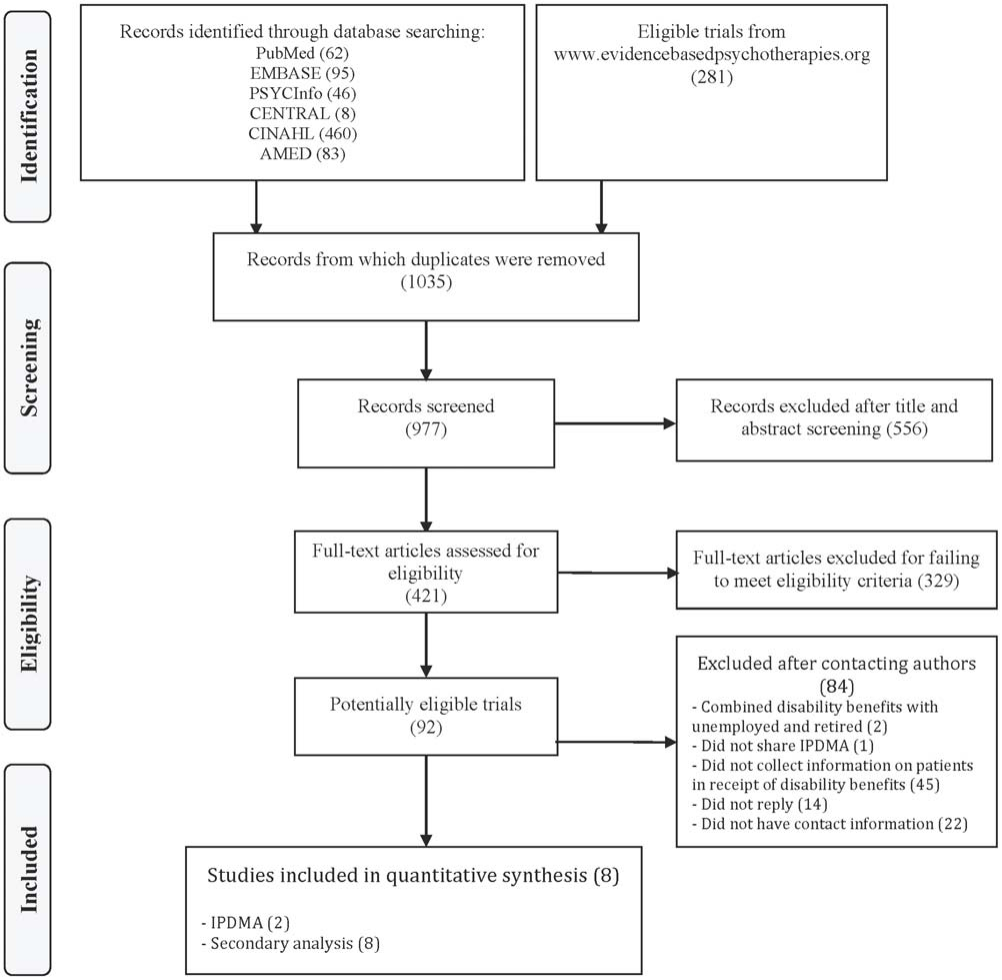
Flow chart of study eligibility.

After establishing author contact for 56 of the 70 trials for which we acquired contact information, we found that 45 trials did not have an eligibility criterion based on disability benefit status or collect information on disability status, 6 trials did not enrol any patients in receipt of disability benefits, and 5 trials enrolled some patients in receipt of disability benefits. Authors of 4 of the 5 trials that included patients in receipt of disability benefits agreed to provide individual patient data. Two of these trials combined patients who were disabled with unemployed and retired individuals and information specific to receipt of disability benefits were uncertain; these trials were therefore excluded from our IPDMA. Our primary analysis consisted of the 2 remaining trials that included some patients in receipt of disability benefits [41], [42], and our secondary analyses consisted of 8 trials, i.e., 6 trials that did not enrol any patients in receipt of disability benefits [43], [44], [45], [46], [47], [48], and 2 trials that included some patients in receipt of disability benefits (Figure 1) [41], [42].

### Study characteristics

Seven studies were parallel group RCTs [41], [42], [43], [44], [45], [46], [47], and one was a cluster RCT [48]. Table 1 describes the characteristics of the 8 eligible trials, and Table 2 provides details regarding their interventions.

**Table 1:** Characteristics of studies.

Study		Sample size		Age (mean ± SD)		Patient Population	Treatment group	Control group	Depression outcomes reported	Included patients on disability benefits
	Country	CBT	Control	CBT	Control					
De Graaf 2009 [41]	The Netherlands	100	103	45.2±10.9	45.1±12.2	Depression	CBT+TAU	TAU	BDI–II	Yes
Dozois 2009 [42]	Canada	25	23	NR	NR	Depression	CBT+pharmaco-therapy	Pharmaco-therapy alone	BDI–II; HRSD	Yes
Naeem 2011 [47]	Pakistan	17	17	32.35±8.9	33.64±1.0	Depression	CBT+pharmaco-therapy	Pharmaco-therapy alone	HADS; BSI	No
Faramarzi 2007 [43]	Iran	42	40	28.3±3.8	28.4±5.3	Depression in infertile women	CBT	Minimal or no treatment	BDI–II	No
Hollon 1992 [44]	USA	25	57	NR	NR	Nonpsychotic, nonbipolar depressed outpatients	CBT+pharmaco-therapy	Pharmaco-therapy alone	BDI; HRSD	No
Miranda 2003 [45]	USA	90	89	29.8±7.9	29.5±9.1	Depression in predominantly low-income young minority women	CBT	TAU	HRSD	No
Misri 2004 [46]	Canada	19	16	29.5±3.3	30.8±5.9	Postpartum depression	CBT+pharmaco-therapy	Pharmaco-therapy alone	HRSD	No
Rahman 2008 [48]	Pakistan	463	440	26.5±5.2	27.0±5.1	Perinatal depression	CBT	TAU	HRSD	No

CBT –Cognitive Behavioural Therapy; TAU – Treatment As Usual; SD – Standard deviation; NR – Not reported; BDI–II – Beck Depression Inventory-II; HRSD – Hamilton Rating Scale for Depression; HADS – Hospital Anxiety and Depression Scale; BSI – Bradford Somatic Inventory.

**Table 2:** CBT details from studies.

Study	Mode of administration of CBT	Duration of CBT per visit	Frequency of CBT	Total duration of CBT	Clinical background of the individuals administering CBT	Was there a standardized program or certification process that CBT providers have undergone or had to undergo?
De Graaf 2009 [41]	Computer/internet based CBT	30 minutes	1 per week	9 weeks	Not reported	Not reported
Dozois 2009 [42]	In-person individualized CBT	1 hour	1 per week	15 weeks	Master's level therapist	Not reported
Naeem 2011 [47]	In-person individualized CBT	Not reported	1 to 2 sessions per week	9 weeks	Psychiatrist; psychology graduates	Not reported
Faramarzi 2007 [43]	In-person group CBT	2 hours	1 per week	10 weeks	Psychologist	Not reported
Hollon 1992 [44]	In-person individualized CBT	50 minutes	2 in the first 4 weeks, 1 or 2 in the next 4 weeks, and 1 in the last weeks	12 weeks	Psychologist; social worker	Not reported
Miranda 2003 [45]	In-person individualized CBT	Not reported	1 per week	8 weeks	Psychologist; psychotherapist	Not reported
Misri 2004 [46]	In-person individualized CBT	1 hour	1 per week	12 weeks	Psychologist	Not reported
Rahman 2008 [48]	In-person individualized CBT	Not reported	4 in 1st month, 3 in 2nd month, and 1 per month for next 9 months	11 weeks	Lady health workers	Not reported

CBT –Cognitive Behavioural Therapy.

### Risk of bias within studies

**Figure 2:**
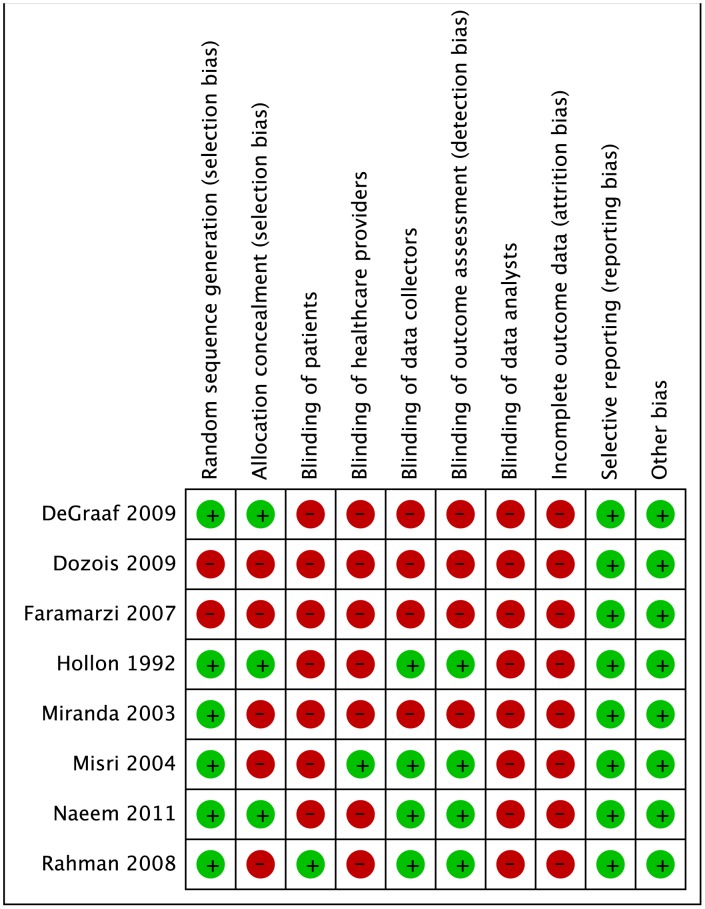
Risk of Bias within studies. ‘+’ denotes low risk of bias, and ‘−’ represents high risk of bias.

Protection against bias was generally poor (Figure 2). All 8 trials reported loss to follow-up (LTFU), ranging from 4% to 40%. Four trials excluded those LTFU and performed a complete case analysis [41], [42], [43], [48], 2 used the last observation carried forward [44], [46], 1 used multiple imputation (56), and 1 did not report an approach [47].

### IPDMA

Two trials including data on patients receiving disability benefits enrolled a total of 227 patients; 34 in receipt of disability benefits and 193 not receiving disability benefits. The mean (SD) baseline BDI–II score for patients with disability benefits was 32.9 (±8.55) and for patients not receiving disability benefits 26.9 (±7.9).

Pooled results from these 2 trials, including both those receiving and not receiving disability benefits, suggested a possible benefit of CBT on depression (MD = −2.61; 95% CI = −5.28 to 0.07; p = 0.06, minimally important difference [MID] = 5), as did results from both the subgroup of patients in receipt of disability benefits (MD = −6.88; 95% CI = −14.06 to 0.31), and patients not receiving disability benefits (MD = −2.22; 95% CI = −5.07 to 0.63). Results suggested a possible larger effect on reducing depression in those receiving versus not receiving disability benefits, though the confidence interval includes a small reduction in benefit in those receiving benefits (MD = −4.46; 95% CI = −12.21 to 3.30; p = 0.26; MID = 5).

### Secondary analyses

There were no significant differences in the effect of CBT on depression among patients not in receipt of disability benefits across studies that enrolled patients receiving disability benefits and studies that did not (p = 0.26) (Figure S1). There were no significant differences in the effect of CBT on depression within patients not receiving disability benefits in studies comparing CBT plus pharmacotherapy versus pharmacotherapy alone (p = 0.94) (Figure S2). There were no significant differences in the effect of CBT on depression within patients not receiving disability benefits in studies comparing CBT versus TAU/standard care (p = 0.59) (Figure S3). Our a priori subgroup hypotheses failed to explain the heterogeneity observed in our secondary analyses.

## Discussion

### Summary of evidence

This is the first systematic review comparing the effect of receiving disability benefits on depression following treatment with CBT. We failed to find differences in the effect of CBT on depression between patients receiving disability benefits and patients not receiving disability benefits. The results suggest a possible greater effect in those receiving disability benefits (−4.46 BDI units in which the minimally important difference is 5), and the boundaries of the confidence interval suggest that if there is a decrement in benefit, that decrement is small (no greater than 3.30 BDI–II units). Nevertheless, these data come from only 34 patients receiving disability benefits, so that any inferences regarding relative effect in the two populations are very weak.

The strengths of our review include a comprehensive and transparent search strategy, independent and duplicate eligibility assessment, use of the most commonly reported instrument with the most established reliability and validity (BDI–II) for our pooled analysis, and use of individual patient data from eligible trials, allowing adjustment for potential confounding predictors. We also ensured rigorous data abstraction by using detailed written instructions, conducting formal calibration exercises, conducting in duplicate, and implementing a consensus approach to resolve disagreement. We contacted authors to verify whether they enrolled patients in receipt of disability benefits and achieved an 80% response rate among trials for which we were able to acquire author contact information.

Although no prior reviews have explored the effect of CBT in patients receiving disability benefits, reviews have explored the effect of compensation in other patient populations. A 2005 systematic review found that the presence of compensation was associated with worse outcome (combination of functional, quality of life, pain and patient satisfaction outcome that was rated as satisfactory or unsatisfactory by review investigators) after surgery [29]. This was consistent with findings from systematic reviews regarding chronic pain and closed-head injuries [49], [50], which reported a significant effect between compensation and poor outcome. This indirect evidence, however, does not address the relative effect of interventions in the populations (one may have poorer outcomes, but still have larger treatment effects if results without treatment are very poor). In the two trials we examined, patients in receipt of disability benefits had a greater severity of depression than those who were not receiving disability benefits (baseline BDI–II of 32.9 versus 26.9). Although a prior review reported that the effectiveness of CBT was reduced in patients with severe depression compared to those with mild to moderate depression [51], we found no suggestion of a smaller effect of CBT in patients receiving disability benefits.

### Limitations

Our study has limitations. First, our IPDMA is based on only 34 patients in receipt of disability benefits and 193 patients not receiving disability benefits. The extent to which findings from this small sample will generalize to a wide population of individuals in receipt of benefits is uncertain. Second, our secondary analyses showed substantial heterogeneity within subgroups of patients not receiving disability benefits, which could not be explained by our *a priori* hypotheses. Possible explanatory factors that we were unable to explore due to limitations in the reporting of trials include baseline severity of depression, duration of depression, frequency of CBT, and experience of CBT providers. Third, none of the trials evaluated the effect of CBT on return to work (RTW), a critical outcome for patients receiving disability benefits and for insurers providing benefits. It remains possible that CBT may improve BDI–II scores, but may not have any effect on claim resolution or RTW. Future trials should include these outcomes in order to ascertain a BDI–II threshold that is associated with RTW and claim resolution.

### Conclusions

If the use of CBT to manage depression among patients receiving disability benefits was less effective than in patients not receiving disability benefits, clinicians and payers might reasonably choose alternative treatment strategies (e.g. pharmacotherapy, other psychotherapies or a combination of both). The limited evidence available, however, provides no support for this hypothesis and suggests that, for the time being, CBT should continue as a recommended approach for addressing depression in patients receiving disability benefits. Secure inference will, however, only be possible after the conduct of much larger comparative trials, conducted with low risk of bias and in collaboration with insurers.

## Supporting Information

Figure S1 **Effect of cognitive behavioural therapy in patients not receiving disability benefits in studies including patients receiving disability benefits versus those that did not.**
(TIF)

Figure S2 **Effect of cognitive behavioural therapy on depression within patients not receiving disability benefits in studies comparing CBT plus pharmacotherapy versus pharmacotherapy alone.**
(TIF)

Figure S3 **Effect of cognitive behavioural therapy on depression within patients not receiving disability benefits in studies comparing CBT versus TAU/standard care.**
(TIF)

Checklist S1 **PRISMA Checklist**
(DOC)
