# Correction: Effectiveness of Cognitive Behavioral Therapy for Depression in Patients Receiving Disability Benefits: A Systematic Review and Individual Patient Data Meta-Analysis

**DOI:** 10.1371/annotation/d2692d0c-26c3-4a7d-b4f5-1139e051e59a

**Published:** 2013-06-14

**Authors:** Shanil Ebrahim, Luis Montoya, Wanda Truong, Sandy Hsu, Mostafa Kamal el Din, Alonso Carrasco-Labra, Jason W. Busse, Stephen D. Walter, Diane Heels-Ansdell, Rachel Couban, Irene Patelis-Siotis, Marg Bellman, L. Esther de Graaf, David J. A. Dozois, Peter J. Bieling, Gordon H. Guyatt

The affiliation for Rachel Couban is incorrect. The correct affiliation is:

Institute for Work and Health, Toronto, Ontario, Canada 

