# Supplementary figures and images for: Effectiveness of Cognitive Behavioral Therapy for Depression in Patients Receiving Disability Benefits: A Systematic Review and Individual Patient Data Meta-Analysis
Source: PLoS One. 2012 Nov 29;7(11):e50202. doi: 10.1371/journal.pone.0050202 (PMC3510249; doi:10.1371/journal.pone.0050202)

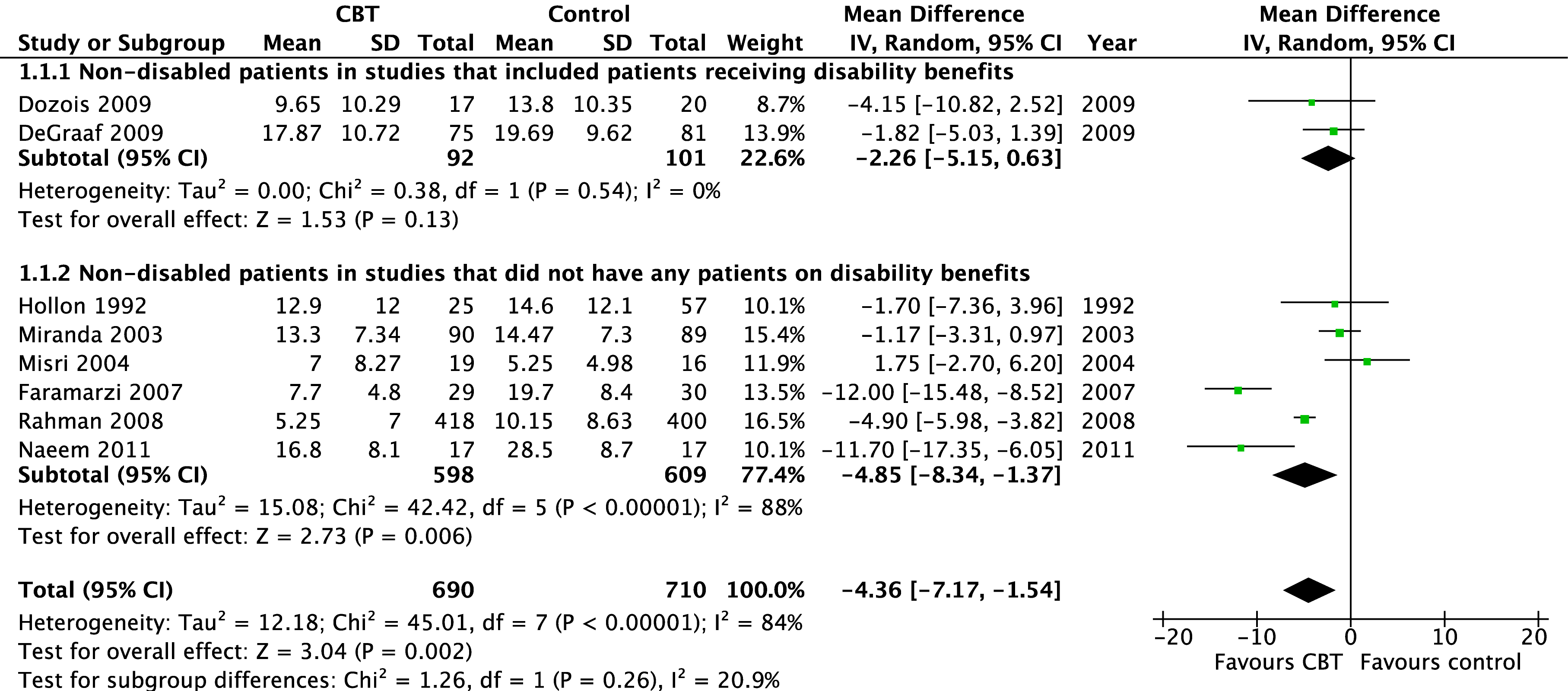

Supplement: Figure S1 — Effect of cognitive behavioural therapy in patients not receiving disability benefits in studies including patients receiving disability benefits versus those that did not. (TIF) [file pone.0050202.s001.tif]

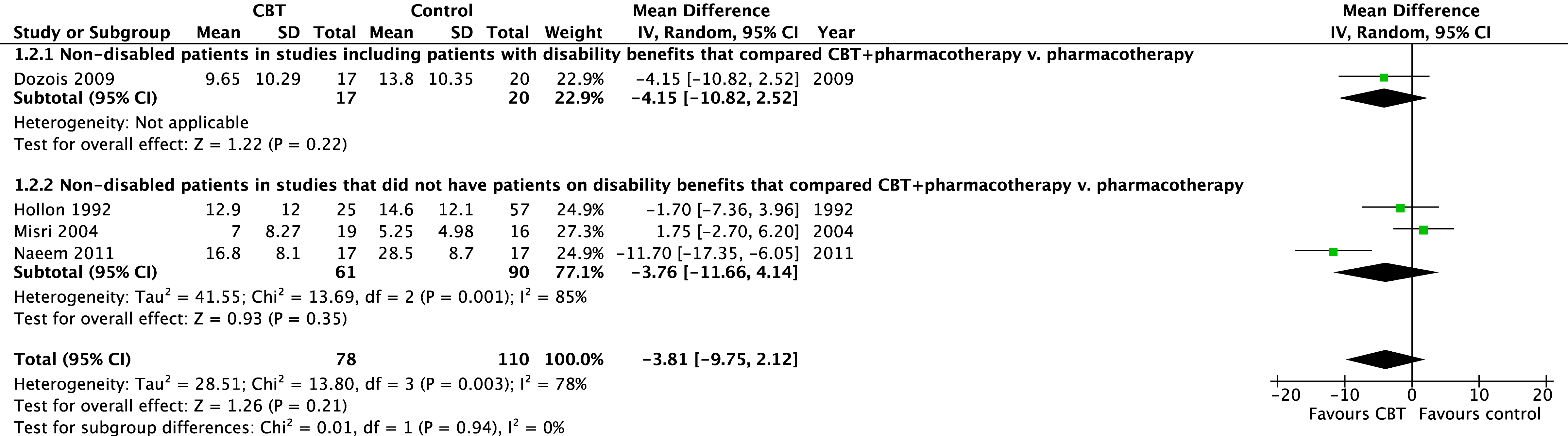

Supplement: Figure S2 — Effect of cognitive behavioural therapy on depression within patients not receiving disability benefits in studies comparing CBT plus pharmacotherapy versus pharmacotherapy alone. (TIF) [file pone.0050202.s002.tif]

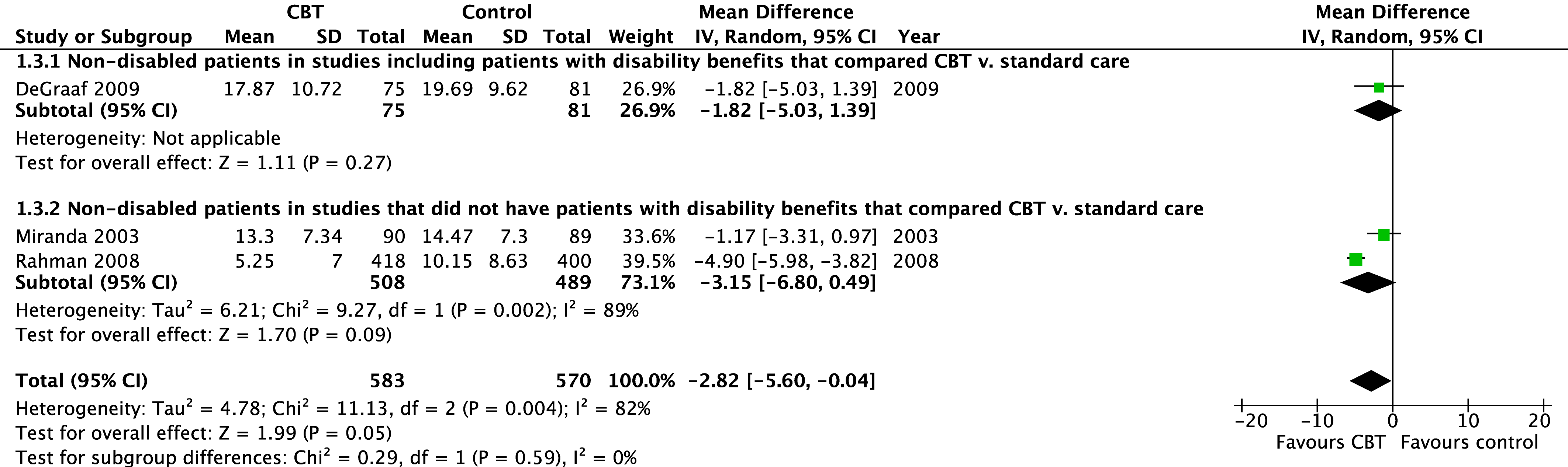

Supplement: Figure S3 — Effect of cognitive behavioural therapy on depression within patients not receiving disability benefits in studies comparing CBT versus TAU/standard care. (TIF) [file pone.0050202.s003.tif]
